# Prevention Science and Health Equity: A Comprehensive Framework for Preventing Health Inequities and Disparities Associated with Race, Ethnicity, and Social Class

**DOI:** 10.1007/s11121-022-01482-1

**Published:** 2023-02-09

**Authors:** Anthony Biglan, Ronald J. Prinz, Diana Fishbein

**Affiliations:** 1 Oregon Research Institute, 2324 West 28th Avenue, Eugene, Eugene, OR 97405, USA; 2 University of South Carolina, Columbia, USA; 3 University of North Carolina, Chapel Hill, USA; 4 The Pennsylvania State University, Pennsylvania, USA; 5 National Prevention Science Coalition to Improve Lives, Pennsylvania, USA

## Abstract

The ultimate goal of our public health system is to reduce the incidence of disability and premature death. Evidence suggests that, by this standard, the USA falls behind most other developed countries largely as a function of disparities in health outcomes among significant portions of the US population. We present a framework for addressing these disparities that attributes them, not simply to differences in the behavioral and physical risk factors, but to social, environmental, and structural inequities such as poverty, discrimination, toxic physical setting, and the marketing of harmful products. These inequities result from de facto and instituted public policies. An analysis of the NIH research portfolio indicates a relative lack of investment in experimental evaluations of preventive interventions—especially studies targeting disadvantaged populations. Moreover, experimental research on reducing social inequities is almost entirely lacking. A line of research focusing on the drivers of inequities and their dissolution must include experimental evaluation of strategies for getting policies adopted that will reduce inequities. In conclusion, a summary is provided of the types of research that are needed and the challenges involved in conducting the experimental research that is essential for reducing inequities and disparities and, in turn, prolonging life.

## Introduction

If the ultimate goal of public health is to prevent disability and premature death, then substantial improvements in our public health system are needed. This paper documents the high levels of premature death in the USA compared with other developed countries and emphasizes the extent to which this is due to our failure to reduce inequities that result in higher rates of death among Black, Indigenous, and poor White populations compared with the population as a whole. These disparities are not simply due to differences in health compromising behaviors. They are also due to inequities in the adverse social determinants of health, which include poverty, discrimination, harmful marketing, poorly maintained schools and neighborhoods, toxic physical environments, and lack of access to quality healthcare (Healthy People 2030, 2021). Progress requires greater investment in experimental research to prevent health-compromising behaviors and conditions by focusing on adverse social determinants of health.

**Fig. 1:**
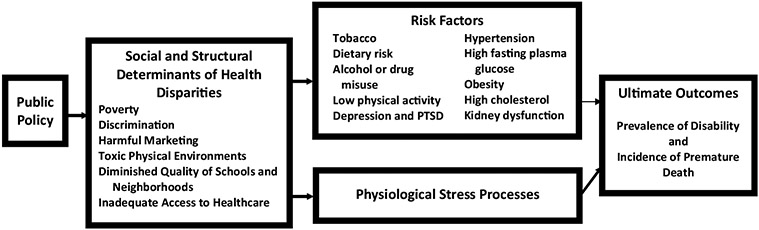
A comprehensive framework for understanding health inequities and disparities

Figure 1 summarizes our analysis. Premature death and the disabilities that cause them are, in part, the result of unhealthful behaviors and physiological conditions, which are themselves influenced by unhealthful behavior. Intertwined with problematic behavior patterns are physiological stress responses that contribute to metabolic syndrome and chronic diseases that shorten lives (Miller et al., 2011). These behaviors and physiological stress responses are, in turn, exacerbated by adverse social determinants of health. The prevalence and persistence of negative social influences are, to a great extent, due to public policies that have eroded the wellbeing of a significant portion of the population over the past fifty years. A comprehensive program of research to reduce health disparities must address all of these influences.

## Disparities in Premature Death

Significant progress has been made in improving the health of Americans in the past seventy years. The age-adjusted death rate per 100,000 people has been cut in half since 1950 (Centers for Disease Control & Prevention, 2018). However, we lag behind other developed nations in prolonging longevity. According to the United Health Foundation, life expectancy at birth in the USA is 78.6 years. We rank 28th out of 36 countries that are members of the Organization for Economic Cooperation and Development.

Importantly, however, life expectancy rates are not equally distributed; there are stark disparities depending on race, ethnicity, and social class. As of 2015, Black people under 65 had higher death rates than Whites for all-cause mortality (Cunningham et al., 2017). The rate of all-cause mortality among Native Americans is 30% higher than the rate for all races (Indian Health Services, 2019) combined. However, disparities in mortality in the Hispanic population are found only among non-white Hispanics (Arias et al., 2020). In addition, there are disparities in death associated with the pandemic, with Black and Latino populations having reductions in life expectancy two to three times those for the White Population (Andrasfay & Goldman, 2022).

Mortality rates also differ by socioeconomic status, with the disparities for low SES on the rise in recent years (Bosworth, 2018). Moreover, mortality rates for Non-Hispanic whites in the USA stopped declining around 2000 and then actually increased (Case & Deaton, 2017). During this same period (2000 to 2014), mortality rates continued to decline significantly in France, Germany, the UK, Canada, Australia, and Sweden. It is imperative that further research is devoted to interventions at all levels—individual, family, community, systems, and policies—to develop best practices that measurably reduce these disparities.

Two other important disparities in life expectancy are beyond the scope of our analysis.

On average, American men die about 5 years earlier than women (Shmerling, 2020), and individuals with developmental disabilities have a lower life expectancy than those without disabilities, with an average age at death of 63 (Lauer & McCallion, 2015).

## Progress and Limitations in Prevention and Treatment Research

Prevention and treatment research have made enormous progress over the past fifty years in developing and evaluating programs and policies that could reduce most of the disabling psychological, behavioral, and physical health problems contributing to premature death. For example, efficacious behavioral treatments have been validated for depression, anxiety, schizophrenia, eating disorders, insomnia, anger, aggression, criminal behavior, smoking cessation, and drug and alcohol problems (Hofmann et al., 2012) as well as obesity (Jacob et al., 2018). Research on family and school preventive interventions has produced numerous strategies capable of making families and schools more nurturing and preventing psychological and behavioral problems of children and adolescents (National Academies of Sciences, Engineering, & Medicine, 2019a). At the same time, policies such as increasing the taxation on tobacco and alcohol (Wagenaar et al., 2010) and raising the drinking age (McCartt et al., 2010) have demonstrated population-level effects.

Progress via controlled studies has demonstrated significant impact on the recipients of these interventions. However, this progress has not been accompanied by population level reductions in incidence and prevalence of many common and costly psychological and behavioral problems. The Centers for Disease Control and Prevention (CDC) reported that “Over a 10-year period, from 2007–2008 to 2015–2016, the percentage of adults with depression did not change significantly” (Brody et al., 2018). Similarly, the CDC concluded that “there is no evidence that the prevalence rates of anxiety disorders have changed in the past years” (Bandelow & Michaelis, 2015). Indeed, suicide rates increased between 1999 and 2018 (Hedegaard et al., 2020). During this same period, drug overdose rates reached historic highs, from less than 20,000 per year to more than 67,000 in 2018 (National Institute on Drug Abuse, 2021). Between 2000 and 2016, alcohol use and binge drinking increased significantly among those over fifty (Grucza et al., 2018). Obesity also increased steadily between 1999 and 2018. In fact, by 2018, the prevalence of obesity in the US population of adults over 20 was 42.4% and the prevalence of severe obesity nearly doubled, from 4.7% to 9.2% (Hales et al., 2020).

There have been some improvements in population health, however, such as the reduction in cigarette smoking (Cornelius et al., 2020) and a significant decline in adolescent drug use (Monitoring, 2021), though this change may be due to a pandemic-related decrease in peer contacts. There have also been significant reductions in juvenile arrests for violent crime since 2006. Finally, the teen pregnancy in the USA has declined significantly, although it is still higher than other industrialized countries (Centers for Disease Control & Prevention, 2021; Osterman et al., 2022).

We have also witnessed progress in the development of efficacious educational programs. Over the past forty years, education researchers have developed numerous instructional strategies that improve academic success and reduce racial and socio-economic disparities in reading skill (Stockard et al., 2018) and mathematics (Gersten et al., 2009; Stockard et al., 2018). In addition, programs have been shown to enhance students’ social, emotional, and behavioral development and prevent multiple problems, such as substance use and antisocial behavior. These approaches include school-based interventions that focus on enhancing classroom and school climate by promoting positive social interactions (Durlak et al., 2010).They also include programs focused on the prevention of specific problems such as cigarette smoking (Thomas, 2006) and other drug use (Tobler & Stratton, 1997), although the effects of the latter programs are generally small.

Here too, progress has not translated into improvements in the prevalence of educational success. The National Assessment of Educational Progress found that, in 2019, only 35% of fourth grade students were proficient in reading, while 36% lacked even basic skills (National Assessment of Educational Progress, 2019). The proportion of Black and Hispanic children who lack basic skills was significantly higher than for the population as a whole (52% and 45% respectively). There has been greater progress in math skills in the past twenty years; however, 19% of 4^th^ grade students lack basic skills in mathematics, with significant disparities according to race and ethnicity (11% for White students, 35% for Black students, and 27% for Hispanic students) (National Center for Education Statistics, 2019). With respect to social and emotional skills, the prevalence of adolescent depression increased significantly between 2005 and 2014 (Mojtabai et al., 2016).

These statistics indicate a relative lack of population level benefits from innovations in prevention. We propose four key reasons for these disappointing trends.

First, research on the dissemination and implementation of evidence-based interventions has yet to result in large increases in adoption and implementation. Across the board—from policymakers to agency administrators and community stakeholders—there is a relative lack of awareness about the knowledge amassed in prevention science or about the efficacious policies and programs that have been developed. As such, embedding and sustaining well-tested interventions has been sluggish to nonexistent in many communities (National Academies of Sciences, Engineering & Medicine, 2019b).

A significant barrier to implementation of evidence-based interventions across school, mental health, and drug treatment settings is that the educational institutions responsible for training teachers and counselors are not required to provide training in evidence-based practices. Education, training, and technical assistance protocols are needed to professionalize the prevention workforce. Teachers, social workers, and other relevant professionals should be able to obtain training through degree programs and continuing education opportunities.

Second, we have not invested sufficiently in experimental evaluations to prevent the leading risk factors for death and disability. A recent analysis of the National Institutes of Health (NIH) research portfolio (Vargas et al., 2019) revealed that the ten leading causes of death account for 74% of deaths, but only 25.9% of NIH-funded projects focused on preventing these causes. In addition, only 34% of grants and 32.5% of funds were awarded to projects studying the prevention of one or more of the ten leading risk factors for death, even though these risk factors have been estimated to account for 57.3% of premature deaths. Moreover, only 2.5% of the total NIH research portfolio in 2019 involved randomized prevention trials. Studies simply documenting the relationship between risk factors and premature death do not sufficiently advance our ability to prevent these putative causes of death to the extent that experimental evaluations of preventive interventions can.

Third, few experimental evaluations of preventive interventions have been conducted in populations experiencing high levels of disparities. The science is clear that the aforementioned risk factors substantially contribute to disparities in death and disability (Dwyer-Lindgren et al., 2017). Despite this, only 0.75% of the total NIH research portfolio of projects in 2019 included a randomized preventive intervention to address one or more of the leading risk factors in a population experiencing health disparities (Murray et al., 2021). Such a low level of commitment to research on the prevention of established risk factors in populations with health disparities makes it unlikely that we will be able to significantly reduce disparities in health and longevity.

However, there is a fourth obstacle to progress: We are failing to address adverse social determinants of health and the public policies that cause or perpetuate them.

## Adverse Social Determinants of Health

The social determinants of health generally refer to social conditions and other contextual influences on health and wellbeing. Accordingly, the emphasis in this paper is on adverse social determinants of health that disproportionately affect certain racial/ethnic groups, socioeconomic strata, and geographic regions. Galea et al. (Galea et al., 2011) searched the English language research literature and estimated the number of deaths in the USA that are attributable to a variety of social factors: “Approximately 245,000 deaths in the United States in the year 2000 were attributable to low education, 176,000 to racial segregation, 162,000 to low social support, 133,000 to individual-level poverty, 119,000 to income inequality, and 39,000 to area-level poverty.” This analysis is consistent with the thesis that it is unlikely we can substantially reduce premature death solely by increasing access to quality health care (Braveman & Gottlieb, 2014). In the sections that follow, we present evidence regarding the role of the social determinants cited by Galea (Galea et al., 2011), as well as harmful marketing, under-resourced schools and communities, and toxic physical environments.

### Child Poverty

Child poverty has remained higher in the USA than in other developed countries (Aber et al., 2012). According to the Annie E. Casey Foundation’s Kids Count, 18 percent of children are living in poverty. While 11% of non-Hispanic Whites and Asians are living in poverty, child poverty is considerably higher for Native American (31%), Black (32%), and Hispanic (26%) children. These numbers have not changed significantly since the year 2000 (Annie E. Casey Foundation, 2018).

### Economic Inequality

Economic inequality has steadily increased over the past fifty years (Alvaredo et al., 2018). Inequality is generally measured in terms of the gap between the income of the top 10% or 20% of the population and the bottom 10 or 20%. The USA has the highest level of inequality of any developed country (Wilkinson & Pickett, 2009). Inequality is a risk factor for a wide variety of psychological and behavioral problems, lower levels of academic success and employment, as well as premature death (Wilkinson & Pickett, 2009).

Evidence indicates that the reason inequality undermines wellbeing is that in an unequal society, people of all levels of income are more likely to have stressful encounters with individuals who are above or below them in the social hierarchy defined by income (Pickett & Wilkinson, 2015).

### Racism and Discrimination

Michelle Alexander (Alexander, 2012) has documented how the rights and wellbeing of a large proportion of the Black population have been eroded over the past fifty years as the war on drugs concentrated law enforcement in minority neighborhoods, increased the penalties for drug possession, allowed no-knock invasion of homes, and disenfranchised as much as 20% of black males in some states. Moreover, discriminatory discipline practices in schools are detrimental to achievement among Black children and heighten risk for involvement in the criminal justice systems (Bell, 2015; Fabelo et al., 2011; Henry et al., 2021).

Discriminatory law enforcement is only one way in which the US society is structured to the disadvantage of Black people and other racial and ethnic groups. Other examples of structural racism include social segregation, which includes residential segregation, segregation in the workplace due to different types of jobs across racial groups, and segregation of social networks. Social segregation contributes to health disparities because it reduces access to high quality health care and increases exposure to slights and micro-aggressions (Gee & Ford, 2011). Moreover, it can deprive people of advantages that derive from knowing influential people in the community, such as lawyers, doctors, and business leaders (Putnam, 2016).

Discrimination is also experienced by Hispanic and Asian people (Lee et al., 2019), as well as other groups such as lesbian, gay, bisexual, and transgendered people (Blake, 2014), Native Americans, (Datz, 2017), and the economically disadvantaged (Equal Justice Under Law, n.d.). Chronic exposure to slights and micro-aggressions contribute to health disparities through their impact on physiological stress responding, which increases the risk of cardiovascular disease (American Psychological Association, 2016; Harrell et al., 2003).

### Toxic Physical Environments

People living in disadvantaged communities are victimized by toxic physical environmental conditions that raise the incidence for example of poisoning, brain damage, and cancers in children and adults. Discriminatory policies and practices that have segregated black people into toxic neighborhood environments have had a devastating impact on the health and well-being of vulnerable communities (Henderson & Wells, 2021). Some of the more notorious injustices include lead paint in dilapidated buildings and housing (LeBrón et al., 2019), contaminated water supply such as that experienced by Flint MI residents (Butler et al., 2016), and placement or continuation of waste dumps and other toxic industrial byproducts in or adjacent to vulnerable communities (Payne-Sturges et al., 2021).

### Marketing of Harmful Products

The impact of harmful marketing is typically overlooked in discussions of social determinants. However, a large number of deaths are attributable to the marketing practices of major industries:

- The tobacco industry: 450,000 deaths per year (Biglan, 2020e)
- The pharmaceutical industry (Biglan, 2020a; Centers for Disease Control & Prevention, 2018): 630,000 deaths due to drug overdose involving prescription opioids or heroin between 1999 and 2016
- The gun industry: 35,000 deaths per year (Biglan, 2020g)
- The alcohol industry: 95,000 deaths per year (Centers for Disease Control and Prevention, n.d.; Pechmann et al., 2011)
- The food industry (Biglan, 2020d)
  Over the past forty years childhood obesity has been increasing (The State of Obesity, 2017)
  One in five American children are obese (U.S. Department of Health & Human Services, 2018)
  The life expectancy of children is now lower than that of their parents (Olshansky et al., 2005)
- The Fossil fuel industry: eight million deaths worldwide due to air pollution (Burrows, 2021) (Biglan, 2020c)

Regulating such sales in light of their impact on health should be a public health goal (Biglan et al., 2019). No company should be able to profit from practices that result in these consequences.

### Disadvantaged Schools and Communities

The Galea et al.’s (2011) analysis attributed the largest number of deaths to low educational attainment. It is well established that schools in high poverty neighborhoods are under-resourced (Boschma & Brownstein, 2016). Disparities in educational outcomes are substantial. For example, with respect to reading proficiency—the academic skill that is foundational for all other academic success—there are large disparities. Across all children, 36% lack even basic skill in third grade; however, the proportion of Black and Hispanic children who lack basic skills is significantly higher (52% and 45% respectively) than for White children (National Assessment of Educational Progress, 2019), and only 19% of Native American fourth graders were proficient readers. We were unable to find data on poor White children; however, only 45% of White children were found to be proficient, and it has been well-established that socio-economic status is a strong correlate of academic failure (Sirin, 2005). These disparities exist despite the fact that effective reading instruction has been shown to eliminate reading disadvantage (Stockard et al., 2020).

Putnam has documented the large gaps in additional resources in disadvantaged communities (Putnam, 2016). For example, compared with affluent neighborhoods where extracurricular activities are available in schools and recreational settings are plentiful, disadvantaged neighborhoods lack activities that would not only broaden young people’s skill and knowledge, but would provide alternatives to experimentation with anti-social behavior.

### Inadequate Access to High Quality Healthcare

Policymakers’ discussions of health disparities have generally given the greatest attention to disparities in access to health care. This is indeed a factor affecting premature death. As many as 35,000 people die each year due to lack of healthcare (Wilper et al., 2009). However, that number pales in comparison to the number of deaths due to social determinants of health. More likely, inadequate healthcare combines with the other adversities to exacerbate their impact or at least fail to mitigate them.

## The Impact of Public Policy on the Social Determinants of Health

The extent to which adverse social determinants undermine health needs to be understood in the context of public policy. Here, we briefly summarize ways in which public policy accounts for the inequitable social conditions driving the health disparities outlined above.

### Child Poverty and Economic Inequality

Policies that contribute to child poverty and economic inequality include the following: (a) declining taxation of the wealthiest over the past fifty years (Hope & Limberg, 2020); (b) lack of regulation for marketing of harmful products and predatory financial practices (Biglan et al., 2019; Biglan, 2020b); and (c) policies that undermine union organizing (Mishel et al., 2020). In addition, policies have been blocked that would have benefited poorer families, such as increasing the minimum wage, providing health insurance, and facilitating access to affordable housing.

#### Racism and Discrimination

We cited above some of the laws that have harmed the Black community, including those that enabled or promoted residential segregation, the war on drugs, laws that enabled discrimination in employment, inferior schools, and community conditions in poorer neighborhoods. In addition, policies that have historically engendered segregation and discrimination in housing are responsible to this day for the displacement and exclusion of Black families from attaining affordable housing, relegating many to poorly equipped schools and limited opportunity to succeed. See Jonathon Metzl’s *Dying of Whiteness* (Metzl, 2019), Heather McGhee’s *The Sum of Us* (McGhee, 2021), and *The 1619 Project* (Hannah-Jones, 2019) for thorough documentation of these points.

Discriminatory policies also harm Native Americans. One example is the legal structure of reservations, where the land is “held in trust” for the tribe by the federal government. This means that individuals in the tribe cannot own the land and therefore cannot get mortgages to build on it (Riley, 2016). Another example is the imposition of an oil pipeline on the lands of the Standing Rock Sioux Tribe in the Dakotas.

LGBTQ rights have expanded with the legalization of same-sex marriages. However, numerous states are passing laws that not only discriminate against members of this community but encourage people to be aggressive toward them (Gender Equality Law Center, n.d.).

Finally, numerous public policies discriminate against poor people. They include policies that allow mortgage-related tax deductions that only benefit home owners and thus discriminate against people who cannot afford to buy a home. They also include court rulings that allow disparities in school support among school districts (Encyclopedia of the American Constitution, n.d.).

#### Toxic Physical Environments

Johansen (2020) has delineated numerous case examples where public policy has contributed to toxic environmental conditions. Further, Wood (2014) has thoroughly documented the failure of environmental law to reduce the myriad ways in which corporate practices have contributed to toxic conditions in communities in the USA. She provides numerous examples of the way in which efforts to reduce industrial pollution have been defeated by the power of well-organized industries.

#### Harmful Marketing

One of the central tenets of free-market economic theory is that regulation of business is harmful. According to this view, regulation increases the costs of goods and services, reduces economic growth, and lowers American’s income. Minimizing regulation has therefore been a central objective of policy-makers who subscribe to this theory. However, as we documented above, our failure to regulate the marketing of tobacco, alcohol, guns, and unhealthful food is implicated in hundreds of thousands of premature deaths.

Biglan et al. (2019) argue that the default assumption that regulation of business is inherently harmful has meant that efforts to regulate specific types of marketing, such as that for cigarettes or pharmaceuticals, must overcome this default assumption. For this reason, Biglan et al. (2019) argue that the default assumption needs to be supplanted by a public health principle: All marketing should be regulated on the basis of its impact on public health. In other words, if a marketing practice can be shown to contribute to illness or death for a large number of people, that practices would be prohibited, and *all* profits from the practice would be confiscated. This contingency would mean that fines for harmful practices could no longer function as simply a cost of doing business.

#### Disadvantaged Schools and Communities

Public policies at the local, state, and federal level allow or support large disparities in the funding of schools. In Pennsylvania, for example, the Lower Merion school district, which serves mostly White students, receives $30,000 more per pupil than Philadelphia, where 86% of students are non-White (Camera, 2019).

These disparities are in part the result of policies that have supported residential segregation by allowing discrimination in lending, as mentioned above. However, the problem was made worse by a Supreme Court decision in the 1970s which ruled that states were under no obligation to reduce disparities in school funding caused by local disparities in tax revenues between more and less affluent districts (Camera, 2019). Such disparities were perpetuated by the failure of the federal government to create policies to ameliorate these inequities.

## Building a Comprehensive Research Agenda

The present analysis underscores the complexity involved in the addressing the multiple intertwined inequities that underlie health disparities. The fact that disparities are the result of chronic exposure to multiple stressors which are, in turn, due to policies and practices that systematically undermine wealth and income and promote unhealthful behaviors suggests that the traditionally dominant methods for prevention research will be insufficient. Here, we discuss the kinds of research that are possible, in the hopes of stimulating a discussion to build an effective agenda. We envision five types of research.

### Observational Studies of Risk Factors

The documentation of risk factors for disparities is foundational for research on reducing disparities. Judging from the analysis of Vargas et al. (2019), research on risk factors for disease appears to be the dominant type of prevention research that is being funded, although it is unclear how much of this work has focused on health disparities. These studies are important for pinpointing targets for interventions that could contribute to the prevention of illness and premature death. We have argued above that such studies need to go beyond the study of proximal risk factors to expand our understanding of the influence of social and structural determinants.

### Experimental Evaluation of Single Prevention Programs

A large body of research has evaluated the impact of single prevention programs. Leslie et al. (2016) have identified sixteen evidence-based family interventions that have resulted from this line of research. Similarly, numerous school-based preventive interventions have been shown to prevent the most common and costly psychological and behavioral problems of children and adolescents. However, in both of these areas, only a small minority of studies have been done in disadvantaged populations. A high priority for this line of research would be to assess the value of such interventions for disadvantaged populations.

### Experimental Evaluation of Multiple Interventions

Less common are studies that experimentally evaluate more than one intervention at the same time. For example, the Communities That Care study randomized communities to implement a family intervention and a school-based intervention or to a no-intervention condition. The intervention communities received technical assistance in establishing a plan for introducing evidence-based family, school, and community programs. The impact of the introduction of multiple evidence-based programs was assessed on youth substance use and delinquency. The relative impact of the overall intervention was assessed (Oesterle et al., 2015), but the design did not allow assessment of the precise impact of individual programs.

### Experimental Evaluation of the Impact of Policies and Modification of Policies

It will be difficult to reduce social inequities unless substantial changes are made to public policy. Prevention science has an important role to play, not only in identifying policies that affect social inequities, but also in studying how policies can be changed (Long et al., 2021).

Experimental research is needed on the impact of policies on inequities. This is an area where interrupted time series designs may be more effective than randomized trials. For example, the effect of policies that limit the marketing of alcohol either by controlling the density of outlets in a community or by curtailing sale to minors could be evaluated via interrupted time-series designs that staggered implementation of these policies across a set of communities (Biglan et al., 2000). Similar designs could be used to assess the impact of policies to reduce harsh treatment of community members or policies designed to increase economic wellbeing. Natural experiments, in which some jurisdictions implement a policy and others do not, are also quite valuable. For example, an analysis of the reductions in youth incarcerations in nine states pinpointed the policy changes that led to these reductions.

Research on changing public policy is in its infancy (Crowley & Jones, 2017). We need to build a body of evidence that pinpoints how policies can be promoted that reduce the most significant inequities. Here too experimental evaluations of strategies are needed.

### Experimental Evaluation of Comprehensive Community Interventions

Although further research evaluating the impact of individual programs and policies will contribute to reducing disparities, more comprehensive community interventions are needed. The research framework created by the National Institute on Minority Health and Health Disparities is consistent with this view (National Institute on Minority Health & Health Disparities, 2017). The evidence we reviewed about the social determinants of health suggests that our impact on health inequities and disparities will be limited, if we do not address the problems of poverty, economic inequality, discrimination, and harmful marketing and improve the quality of neighborhood environments.

Comprehensive community interventions would use well-established community organizing principles (Watson-Thompson et al., 2008) to assist community members in addressing the issues in their community that they feel should have the highest priority. Prevention strategies would likely include implementing evidence-based family and school programs. But they would also address problems like harmful marketing, poverty, toxic police practices, economic development, and under-resourced schools.

Such interventions pose significant challenges to obtaining empirical evidence about their effectiveness. The existence of multiple efforts to address a neighborhood’s or community’s problems makes it difficult to tease out which components of the intervention are effective and which are not. A randomized trial of communities, while feasible, could not ensure that the same strategies were used in all communities because a key principle is that each community directs the selection and implementation of specific efforts to best meet that community's needs.

Consequently, it might prove difficult to convince NIH study sections to accept such community-driven implementation designs. For this reason, a robust discussion of the issues involved in developing effective experimental methods for assessing and refining comprehensive community interventions is sorely needed. If we do not devise effective methods for evaluating such interventions, there will continue to be a divide between research and practice. This disconnection impedes the adoption of evidence-based interventions and fails to strengthen the impact of efforts by local governments and philanthropic organizations to reduce the many inequities and disparities that undermine wellbeing in the United States.

## A Thoroughgoing Public Health Approach to Disparities

An implicit assumption of the prevention science community is that the wider adoption of experimentally evaluated programs, policies, and practices will help to reduce health disparities (Fagan et al., 2019). To that end, it is imperative that far more funding goes to the kinds of studies we just described.

However, addressing the many inter-connected problems affecting health requires a much broader mobilization of society than can be achieved through the research-to-practice pipeline alone. Putnam and Garrett (Putnam & Garrett, 2020) have documented the social movement that occurred in the first half of the twentieth century, its contribution to the expansion of the middle class, and the decline in social solidarity and wellbeing in the second half of the century that was largely driven by advocacy for free market economic theory (Biglan, 2020f). They call for a social movement to reverse the changes that have occurred.

This perspective calls for a far greater focus on altering public policy. So long as de facto policies persist for child poverty, economic inequality, unregulated marketing, and structural racism, even a substantial increase in the adoption of evidence-based interventions will not eliminate the inequities that underlie disparities in the health and wellbeing of Americans. The prevention science community has a critical role to play in educating civic and public health leaders about evidence-based programs and policies. But we must also address the larger cultural and political factors that continue to impede the realization of a society in which every person lives a healthy and productive life in caring relations with others (Biglan, 2020f).
